# Effect of Cooling Rate on Phase and Crystal Morphology Transitions of CaO–SiO_2_-Based Systems and CaO–Al_2_O_3_-Based Systems

**DOI:** 10.3390/ma12010062

**Published:** 2018-12-25

**Authors:** Mei Leng, Feifei Lai, Jiangling Li

**Affiliations:** 1 College of Materials Science and Engineering, Chongqing University, Chongqing 400044, China; 201809021083@cqu.edu.cn (M.L.); 20142953@cqu.edu.cn (F.L.); 2 Chongqing Key Laboratory of Vanadium-Titanium Metallurgical and New Materials, Chongqing University, Chongqing 400044, China

**Keywords:** phase, crystal morphology, cooling rate, mold flux

## Abstract

The phase and crystal morphology transitions of two typical types of mold fluxes were investigated fundamentally using differential scanning calorimetry (DSC) and confocal scanning laser microscopy (CSLM) techniques. For the traditional CaO–SiO_2_–CaF_2_-based mold flux, different cooling rates can change the phases and the crystal morphologies. Faceted cuspidine and CaSiO_3_ are co-precipitated when the cooling rate is less than 50 °C·min^−1^. The phases transform from Ca_4_Si_2_O_7_F_2_ and CaSiO_3_ to Ca_4_Si_2_O_7_F_2_ at the cooling rate of 50 °C·min^−1^. Cuspidine shows four different morphologies: faceted shape, fine stripe, fine stripe dendrite, and flocculent dendrite. The crystalline phases of CaAl_2_O_4_ and Ca_3_B_2_O_6_ are co-precipitated in the CaO–Al_2_O_3_-based mold flux. Neither the phases nor the crystal morphologies change in the low cooling rate range (5 °C·min^−1^ to 50 °C·min^−1^). With decreasing temperature, the morphology of CaAl_2_O_4_ firstly becomes dendritic, and then the dendritic quality gradually changes to a large-mesh blocky shape at the cooling rates of 100 °C·min^−1^, 200 °C·min^−1^, and 500 °C·min^−1^. Different cooling rates do not show an obvious impact on the morphology transition of CaAl_2_O_4_. The strong crystallization ability and large rate of crystallization affect the control of the heat transfer of the CaO–Al_2_O_3_-based mold flux during casting. The big morphology difference between primary crystals of the CaO–SiO_2_–CaF_2-_based mold flux and the CaO–Al_2_O_3_-based mold flux is probably one of the biggest factors limiting lubrication between the CaO–Al_2_O_3_-based mold flux and high-Al steel during casting.

## 1. Introduction

Mold fluxes, as one of the most important additives in the steelmaking process, play an indispensable role in continuous casting. During casting, molten fluxes infiltrate between the solidified shell and the copper mold. This has two main functions: one is to maintain good lubrication between the steel shell and the copper mold, and the other is to control the heat transfer from the steel shell to the mold. The functions of heat transfer and lubrication are deeply related to the surface quality of steel billets, especially at the initial stage of solidification during casting [1,2,3].

Mold fluxes form a slag film consisting of a liquid layer and solid layers between the solidifying steel shell and the copper mold. The solid layers include crystalline and glassy mold fluxes. The distribution and structure of the solid layers affect the control of the lubrication and heat transfer of the mold flux. As previously reported [4,5], the thermal resistance between the copper mold and the solidifying steel shell increases with increasing thickness of the solid slag film. The thickness of the solid layers is closely related to the crystallization behaviors. Many reports have shown that crystallization can reduce heat flux across the mold flux layer [6,7,8]. Hanao et al. [8] found that the reduction in heat transfer was caused by the formation of crystals of cuspidine in the flux film. Additionally, crystallization has a great effect on lubrication. It has been reported that the precipitation of crystals with a large size may decrease the lubrication ability of mold fluxes [9]. Therefore, proper crystallization behaviors are very important for better control of the heat transfer and lubrication properties of the mold flux.

The crystallization behaviors of a mold flux mainly include crystallization ability, crystallization temperature, crystal fraction, and crystal phase and morphology. Previous studies have focused on crystallization ability and temperature [10,11,12]. Consideration of the phase and crystal morphology transitions has been limited. Cho et al. [13] found that the size of the precipitated crystals affected the lubrication of the mold flux. Guo et al. [9] reported that the huge differences in crystal morphology between lime–silica-based mold fluxes and lime–alumina-based mold fluxes may be responsible for the poor lubrication performance of lime–alumina-based mold fluxes. In order to better control heat transfer and lubrication, it is necessary to investigate the changes in the phase and crystal morphology transitions of mold fluxes.

The cooling rate is the one of the most important factors affecting the crystallization behaviors of slag. Guo et al. [9] showed that the crystal morphology of a mold flux is highly related to supercooling. Zhang et al. [14] investigated the crystallization of a mold flux using temperature confocal scanning laser microscopy and found that the precipitated phase and crystal morphology had changed. Furthermore, Yoshiaki Kashiway et al. [15] reported that the liquid slag is exposed to different cooling paths, which can promote or prevent crystallization. Generally, high cooling rates are prone to generating glassy mold fluxes. Conversely, low cooling rates result in crystalized slags. Therefore, it is important to analyze the effect of the cooling rate on the phase and crystal morphology transitions of mold fluxes. There are many methods that have been employed to study crystallization [14,16,17], such as differential scanning calorimetry (DSC), the double/single hot thermocouple technique (D/SHTT), confocal scanning laser microscopy (CSLM), and so on. To fill the gap due to the limited studies on crystal phases and morphologies, the effect of the cooling rate on the phase and crystal morphology transitions of two typical mold fluxes was identified in the present work.

A synthesized conventional CaO–SiO_2_–CaF_2_-based mold flux and a nonreactive CaO–Al_2_O_3_-based mold flux were investigated. The former is extensively used in steel production because of its good performance. The latter is regarded as a promising slag for high-[Al] steel casting [13]. However, the nonreactive CaO–Al_2_O_3_-based mold flux is not used in actual production because of its poor performance. Crystallization behavior is one of the most important factors leading to poor performance of mold flux during casting. In the present work, we focused on investigating the phase and crystal morphology transitions of a conventional CaO–SiO_2_–CaF_2_-based mold flux and a nonreactive CaO–Al_2_O_3_-based mold flux at different cooling rates. A CSLM technique was used to analyze the crystallization behaviors at high cooling rates (100–500 °C·min^−1^) because it is easy to achieve a high cooling rate. DSC can determine the number of phases through the number of exothermic peaks present; this was used to analyze the crystallization behaviors at low cooling rates (5–50 °C·min^−1^).

## 2. Materials and Method

### 2.1. Sample Preparation

A traditional CaO–SiO_2_–CaF_2_-based mold flux and a nonreactive CaO–Al_2_O_3_-based mold flux were synthesized. Reagent-grade powders of CaCO_3_, Na_2_CO_3_, H_3_BO_3_, CaF_2_, SiO_2_, and Al_2_O_3_ were used as the raw materials. In order to obtain CaO, CaCO_3_ was calcined at 1373 K (1100 °C) in a muffle furnace (Nuobadi Corporation, Zhengzhou, China) and held for 8 h; it was identified by calculating the weight loss before and after the experiment. The chemical compositions of the investigated mold fluxes are listed in Table 1.

**Figure 1:**
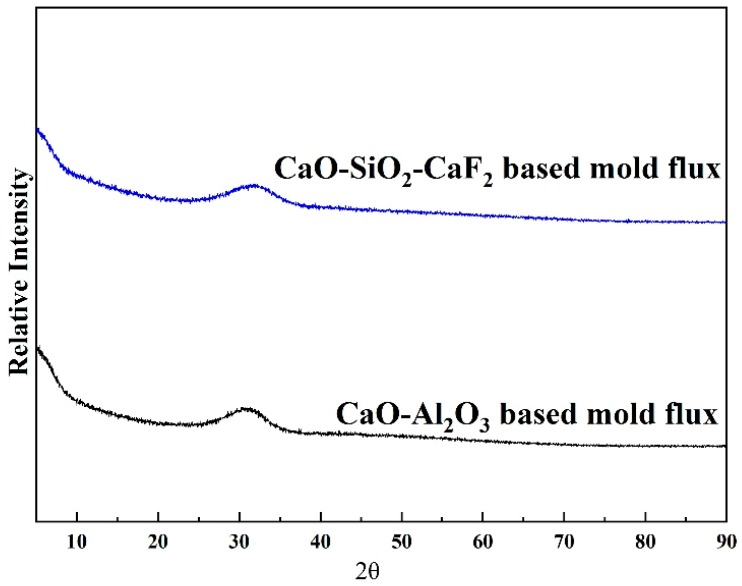
X-ray diffraction patterns of glassy mold fluxes of a CaO–SiO_2_–CaF_2_-based mold flux and a CaO–Al_2_O_3_-based mold flux.

The glassy samples were prepared by the conventional melting and water-quenching method. The mixed sample was placed in a platinum crucible and melted in a high-temperature furnace with molybdenum silicide as the heating element at approximately 1623 K (1350 °C). In order to achieve complete melting and homogenization, the mold flux samples were held at 1623 K (1350 °C) for 2 h. Then, they were quenched with water. The quenched samples were characterized by X-ray diffraction (XRD), as shown in Figure 1. Both exhibited amorphous properties.

### 2.2. Differential Scanning Calorimetry (Cooling Rate: 0–50 °C·min^−1^)

The glassy samples were pulverized into powder for differential scanning calorimetry analysis (DSC) with cooling rates ranging from 0 °C·min^−1^ to 50 °C·min^−1^. The differential scanning calorimetry analysis was performed in an argon atmosphere on each of the glassy samples over the range of 773–1723 K (500–1350 °C) using a Netzsch DSC404 F3 calorimeter (Netzsch Corporation, Selb, Germany). Alfa–Al_2_O_3_ was used as a reference material for the DSC experiments.

### 2.3. Confocal Scanning Laser Microscopy (Cooling Rate: 100–50 °C·min^−1^)

In situ observation of crystallization was performed using a halogen lamp heating stage and a confocal scanning laser microscope (CSLM, VL2000DX, Lasertec Corporation, Yokohama, Japan). The platinum crucible containing the samples was rapidly heated by the light irradiated by the halogen lamp. In the cooling process, the crystallization phenomena were recorded by in situ observation of the surface of the slags. After the sample reached thermal equilibrium, the melt was cooled at a designated rate, and video images of the molten slag were collected during the non-isothermal stage. The nucleation and growth process of the crystals in the investigated mold fluxes were observed during continuous cooling of the slag at fixed rates from 100 to 500 °C·min^−1^.

### 2.4. Phase and Crystal Morphology Identification

The crystalline phase and crystal morphology of the samples after DSC and CSLM measurements were identified by X-ray diffraction (XRD) and a scanning electron microscope equipped with energy-dispersive X-ray spectroscopy (SEM–EDS) for microanalysis. Powder X-ray diffraction measurements were carried out on an 18KW X-ray diffractometer (RIGAKU TTR III, Rigaku Corporation, Tokyo, Japan). SEM examinations were carried out using a TESCAN VEGA 3 LMH (TESCAN Corporation, Brno, Czech Republic) equipped with EDS.

## 3. Discussion and Results

### 3.1. Effect of Cooling Rate on the Phase and Crystal Morphology Transitions of the CaO–SiO_2_–CaF_2_-Based Mold Flux

**Figure 2:**
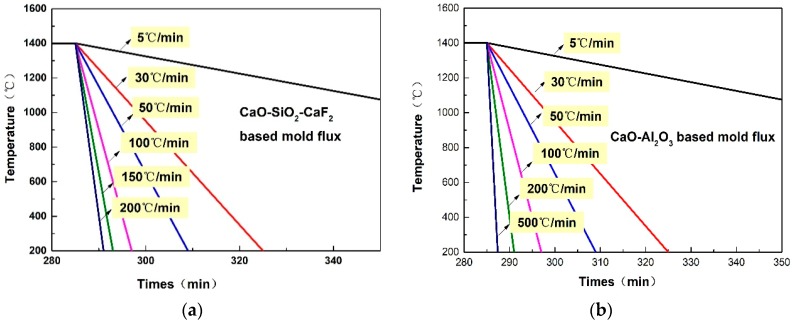
A diagram of the cooling process at different cooling rates in (**a**) a CaO–SiO_2_–CaF_2_-based mold flux and (**b**) a CaO–Al_2_O_3_-based mold flux.

**Figure 3:**
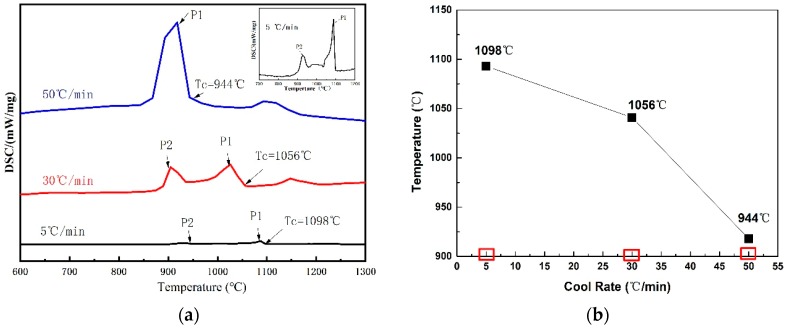
(**a**) Differential scanning calorimetry (DSC) curves of the non-isothermal crystallization of CaO–SiO_2_–CaF_2_-based mold fluxes at the cooling rates of 5 °C·min^−1^, 30 °C·min^−1^, and 50 °C·min^−1^. (**b**) The crystallization temperatures at the cooling rates of 5 °C·min^−1^, 30 °C·min^−1^, and 50 °C·min^−1^.

The crystallization behaviors of the CaO–SiO_2_–CaF_2_-based mold flux were investigated at different cooling rates using DSC and CSLM. The specific cooling rates are shown in Figure 2a. The DSC curves of non-isothermal crystallization of CaO–SiO_2_–CaF_2_-based mold fluxes at the cooling rates of 5 °C·min^−1^, 30 °C·min^−1^, and 50 °C·min^−1^ are shown in Figure 3a. It can be seen that there were two exothermic peaks on the DSC curves at the cooling rates of 5 °C·min^−1^ and 30 °C·min^−1^, and only one exothermic peak at the cooling rate of 50 °C·min^−1^. This indicated the presence of two successive crystallization events for the CaO–SiO_2_–CaF_2_-based mold flux at the cooling rates of 5 °C·min^−1^ and 30 °C·min^−1^, and only one crystallization event occurring at the cooling rate of 50 °C·min^−1^. Additionally, it was observed that the exothermic peaks on the DSC curves moved toward a lower temperature and the shape of exothermic peak became sharper with the increasing cooling rate. This may be attributed to the fact that the nucleation and growth rate of crystals are functions of viscosity and undercooling. Viscosity increases quickly under a high cooling rate, and a stronger driving force is required to initiate the mold flux nucleation [18]. The specific change in the crystallization temperature is shown in Figure 3b. It can be concluded that the crystallization temperature decreased with increasing cooling rate in the CaO–SiO_2_–CaF_2_-based mold flux. The crystallization behaviors of the CaO–SiO_2_–CaF_2_-based mold flux were previously investigated by SEO et al. [19] by employing a DSC technique. They also found similar behaviors in the crystallization.

**Figure 4:**
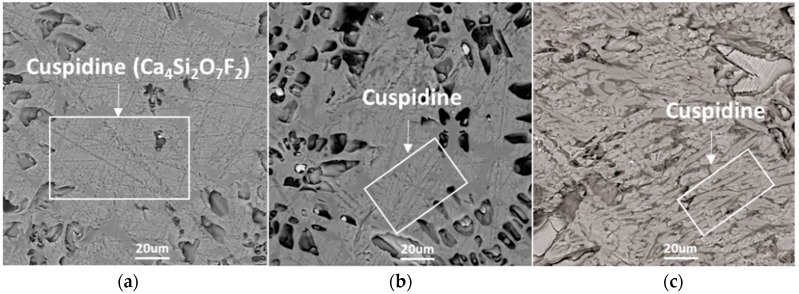
Scanning electron microscope (SEM) micrographs of the CaO–SiO_2_–CaF_2_-based mold flux after DSC measurements: (**a**) 5 °C·min^−1^, (**b**) 30 °C·min^−1^, and (**c**) 50 °C·min^−1^.

In order to identify the phases and crystal morphologies, the samples taken from the DSC measurements were characterized by SEM–EDS, as shown in Figure 4. The main phase was identified as cusipdine (CaO·2SiO_2_·CaF_2_) at all cooling rates. According to the phase equilibrium study on the CaO–CaF_2_–SiO_2_–Al_2_O_3_ flux system carried out by the present author [20], cuspidine, as the primary crystal in mold fluxes with basicity higher than R = 1 (R = CaO mass%/SiO_2_ mass%), was firstly formed. The main morphology of cuspidine was faceted at the cooling rates of 5 °C·min^−1^ and 30 °C·min^−1^. When the cooling rate increased to 50 °C·min^−1^, the morphology of cuspidine changed to fine stripe. It can be concluded that a high cooling rate could induce a morphology transition in the crystallized cuspidine. In addition, the phases decreased with the increasing cooling rate.

**Figure 5:**
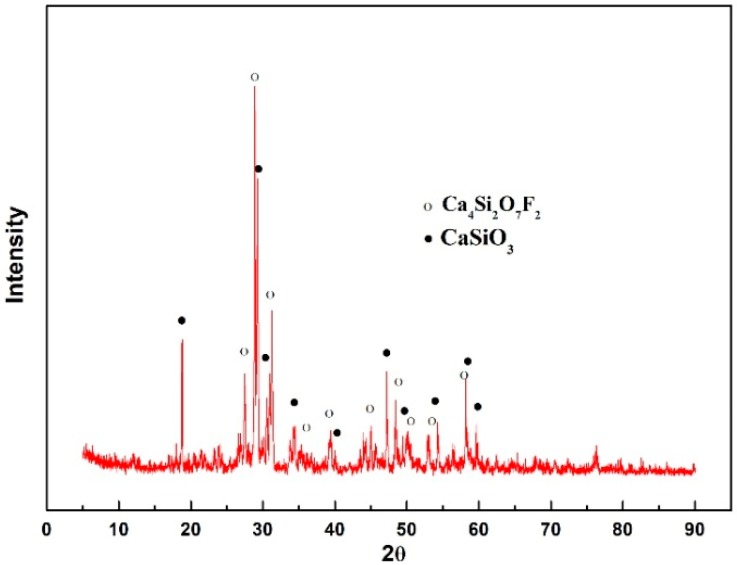
X-ray diffraction (XRD) patterns of the CaO–SiO_2_–CaF_2_-based mold flux at the cooling rate of 5 °C·min^−1^.

Because the phase identification by SEM–EDS after the DSC measurements of the CaO–SiO_2_–CaF_2_-based mold flux could not determine the corresponding crystalline phases of the two exothermic peaks, heat treatment experiments were carried out to analyze the crystalline phase precipitation in the mold fluxes at the cooling rate of 5 °C·min^−1^. After the heat treatment experiments, the samples were subjected to XRD analysis to identify the specific crystalline phase. The results are shown in Figure 5. It was seen that cuspidine and CaSiO_3_ co-precipitated in the CaO–SiO_2_–CaF_2_-based mold flux. The fact that only cuspidine was observed in SEM may be because the second phase, that is, CaSiO_3_, precipitated at a lower temperature with a small crystal size.

**Figure 6:**
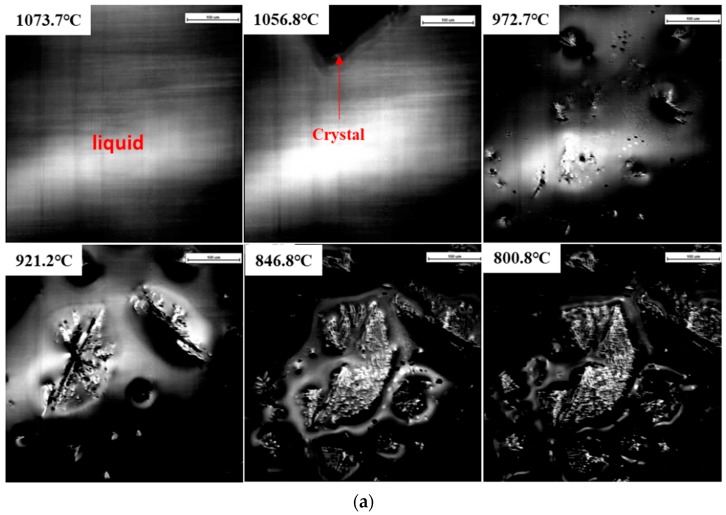

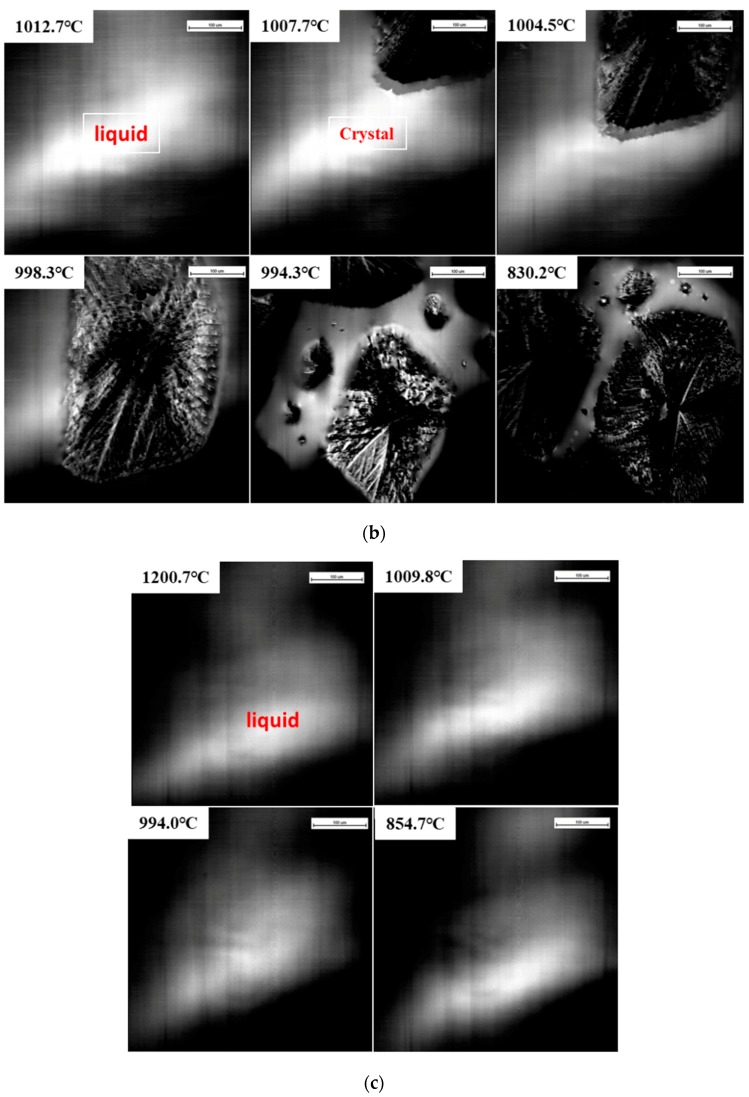
The crystallization process of the CaO–SiO_2_–CaF_2_-based mold flux measured by confocal scanning laser microscopy (CSLM) at different cooling rates: (**a**) 100 °C·min^−1^, (**b**) 150 °C·min^−1^, and (**c**) 200 °C·min^−1^.

**Figure 7:**
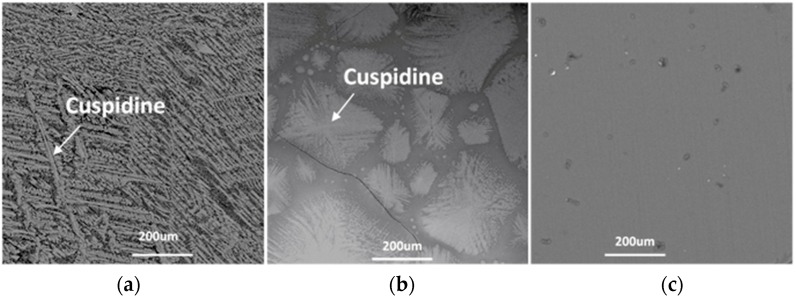
SEM micrographs of the CaO–SiO_2_–CaF_2_-based mold fluxes after confocal scanning laser microscopy (CSLM) measurements at different cooling rates: (**a**) 100 °C·min^−1^, (**b**) 150 °C·min^−1^, and (**c**) 200 °C·min^−1^.

The effect of high cooling rates on the phase and crystal morphology transitions of the CaO–SiO_2_–CaF_2_-based mold flux was analyzed by CSLM. Figure 6a–c show the crystallization process of slag at the cooling rates of 100 °C·min^−1^, 150 °C·min^−1^, and 200 °C·min^−1^, respectively. As we know, liquid fluxes present as a bright white field in CSLM experiments. It was observed that the primary phase precipitated from the molten fluxes, then started to develop, and the liquid phase gradually reduced as the temperature gradually decreased. When the cooling rate was increased to 200 °C·min^−1^, there was no crystallization observed throughout the whole cooling process. The onset of the nucleation and growth of primary crystals is shown in Figure 6a,b. It was observed that the morphology of cuspidine was the blocky shape. When the temperature gradually decreased, distinct dendrites were observed in the blocky-shaped crystal. It was further confirmed by the SEM results in Figure 7a that cuspidine presented fine stripe dendrites at the cooling rate of 100 °C·min^−1^. This is similar to the morphology of cuspidine at the cooling rate of 50 °C·min^−1^. When the cooling rate was increased to 150 °C·min^−1^, the morphology of the cuspidine transformed to the flocculent dendrite shape.

It can be concluded from the DSC and CSLM results that different cooling rates can change the phase and crystallization morphology of a CaO–SiO_2_–CaF_2_-based mold flux. The phases transform from Ca_4_Si_2_O_7_F_2_ and CaSiO_3_ to Ca_4_Si_2_O_7_F_2_ as the cooling rate increases from 30 °C·min^−1^ to 50 °C·min^−1^. Cuspidine shows four different morphologies: faceted shape, fine stripe, fine stripe dendrite, and flocculent dendrite. Many researchers have studied the morphology of crystal [2,9,18,21,22,23,24]. Lu et al. [21] studied the crystal morphology transitions relating to different supercooling degrees of Ni–Si alloy melt and found that the crystal morphology of the alpha-Ni phase transformed from non-faceted to faceted in morphology when the supercooling was larger than approximately 390 °C, while it tended to be dendritic again when the supercooling increased to around 500 °C. Orrling et al. [23] investigated the crystallization of slags using a double hot thermocouple technique and found that the crystal morphology was deeply related to the experimental temperature. They found four different morphologies of crystals (equiaxed crystal at high temperature, columnar crystals from thermocouple towards center, faceted crystals, and fine crystals) based on the melting undercooling. The crystallization process is very complex and is controlled by nucleation and crystal growth. The crystal morphology is mainly governed by the crystal growth process after nucleation [9,25]. There are two important factors that affect crystal growth: one is the elements diffusing from the melt bulk to the crystal–melt interface, and the other is the chemical reactions that occur at the interface. If the crystal growth is controlled by chemical reactions, then the crystal morphology is more likely to be faceted, while it will tend to be dendritic if the crystal growth is determined by the element diffusion process. The chemical reaction rate is proportional to the driving force, and the element diffusion rate is inversely proportional to the viscosity of the melts [9,25]. This indicates that the crystal morphology transition is influenced by the supercooling degree. When the supercooling degree increases, the controlling step alters from chemical reaction at the interface to element diffusion at the interface, because the viscosity of the melt increases greatly with increasing supercooling. This phenomenon can explain the present results concerning the transition of crystal morphology from faceted to dendritic with the increasing cooling rate.

### 3.2. Effect of Cooling Rate on Phase and Crystal Morphology Transitions of the CaO–Al_2_O_3_-Based Mold Flux

**Figure 8:**
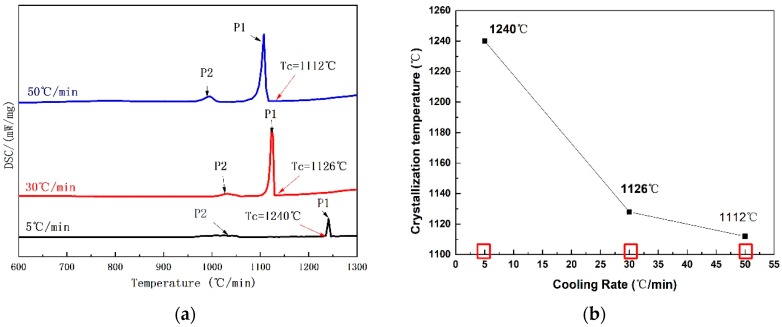
(**a**) DSC curves of non-isothermal crystallization of the CaO–Al_2_O_3_-based mold flux at the cooling rates of 5 °C·min^−1^, 30 °C·min^−1^, and 50 °C·min^−1^. (**b**) The crystallization temperatures at the cooling rates of 5 °C·min^−1^, 30 °C·min^−1^, and 50 °C·min^−1^.

**Figure 9:**
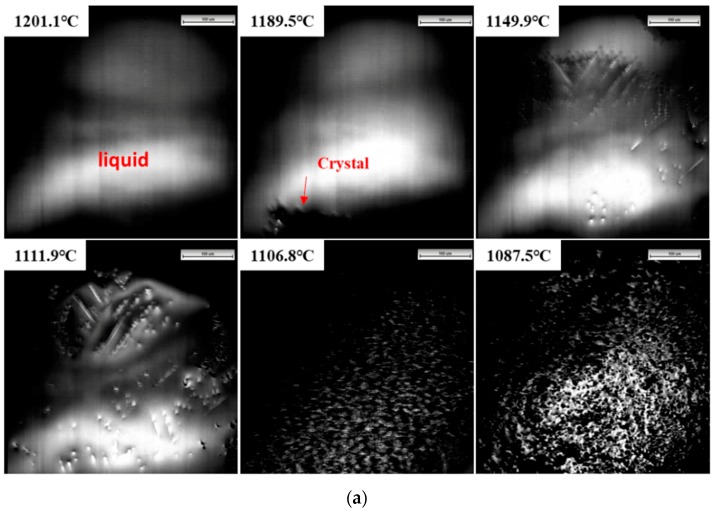

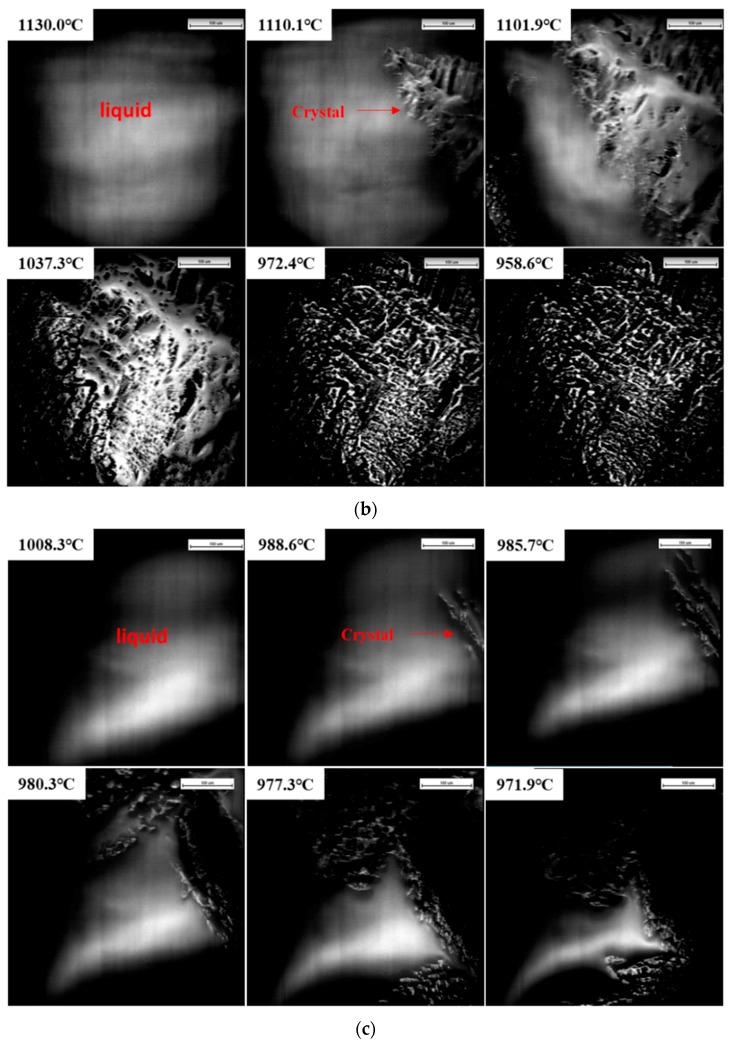
The crystallization process of the CaO–Al_2_O_3_-based mold flux measured by CSLM at different cooling rates: (**a**) 100 °C·min^−1^, (**b**) 300 °C·min^−1^, and (**c**) 500 °C·min^−1^.

**Figure 10:**
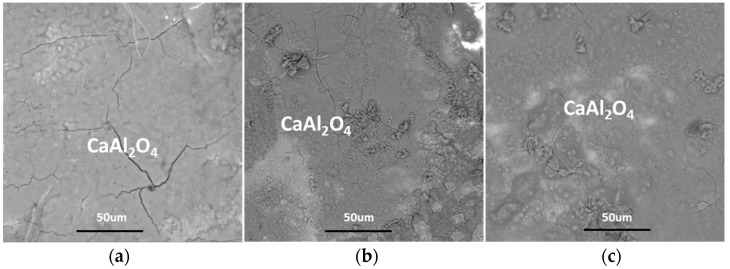
SEM micrographs of the CaO–Al_2_O_3_-based mold flux after DSC measurements: (**a**) 5 °C·min^−1^, (**b**) 30 °C·min^−1^, and (**c**) 50 °C·min^−1^.

**Figure 11:**
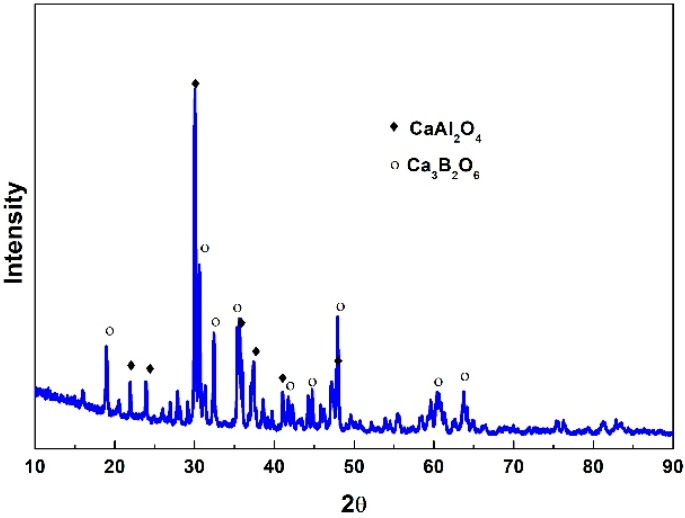
XRD patterns of the CaO–Al_2_O_3_-based mold flux at the cooling rate of 5 °C·min^−1^.

**Figure 12:**
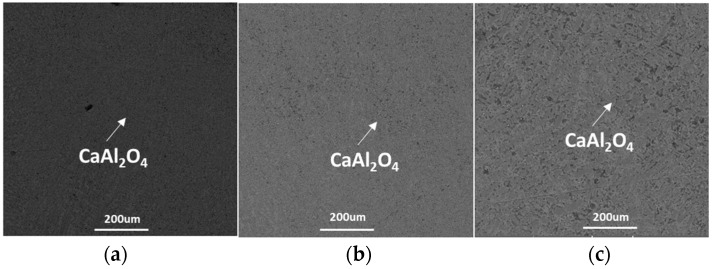
SEM micrographs of the CaO–Al_2_O_3_-based mold flux after CSLM measurements: (**a**) 100 °C·min^−1^, (**b**) 300 °C·min^−1^, and (**c**) 500 °C·min^−1^.

The crystallization behaviors of a CaO–Al_2_O_3_-based mold flux were investigated at different cooling rates using DSC and CSLM. The specific cooling rates are shown in Figure 2b. The DSC and CSLM results are shown in Figure 8 and Figure 9, respectively, and the corresponding XRD and SEM–EDS analysis results are shown in Figure 10, Figure 11 and Figure 12. It can be seen in Figure 8a that there were two exothermic peaks on the DSC curves for samples at different cooling rates. This indicated the presence of two successive crystallization events for the CaO–Al_2_O_3_-based mold flux at different cooling rates. The crystallization temperatures were determined and are shown in Figure 8b. It can be seen that the crystallization temperature decreased from 1240 °C to 1112 °C as the cooling rate increased from 5 °C·min^−1^ to 50 °C·min^−1^. SEM–EDS combined with XRD analysis showed that CaAl_2_O_4_ and Ca_3_B_2_O_6_ co-precipitated. The crystal morphology of the samples identified by SEM–EDS after DSC measurements is shown in Figure 10. The primary phase was identified as CaAl_2_O_4_ at all cooling rates. Because the second phase, that is, Ca_3_B_2_O_6_, precipitated at a lower temperature with a small crystal size, it could not be detected. The main morphology of CaAl_2_O_4_ was a big blocky shape, and it did not change at cooling rates between 30 °C·min^−1^ and 50 °C·min^−1^. Shi et al. [26] investigated the crystallization behaviors of a CaO–Al_2_O_3_-based mold flux and found that the precipitated calcium aluminate crystals are the primary phase and appear as a blocky shape. This is similar to our present results. It can be concluded that the phases and crystal morphologies are not altered by cooling rates between 30 °C·min^−1^ and 50 °C·min^−1^.

The CSLM results showed that the morphology of CaAl_2_O_4_ was first dendritic, and then the dendritic morphology gradually changed to a large-mesh blocky shape at the cooling rates of 100 °C·min^−1^, 200 °C·min^−1^, and 500 °C·min^−1^. The whole crystallization process was extremely rapid. This rapid crystallization process indicated that the crystallization ability of the CaO–Al_2_O_3_-based mold flux is very strong. Different cooling rates did not show an obvious impact on the morphology transition of CaAl_2_O_4_.

Compared with that of the CaO–SiO_2_–CaF_2_-based mold flux, the crystallization temperature of the CaO–Al_2_O_3_-based mold flux was higher. This indicated that the crystal growth of the CaO–Al_2_O_3_-based mold flux started at a higher temperature, so the crystallization was stronger than that of the CaO–SiO_2_–CaF_2_-based mold flux. Many references have reported that the CaO–Al_2_O_3_-based mold flux has a stronger crystallization ability than the CaO–SiO_2_-based mold flux [13,27]. Our result is in agreement with these previous reports. The strong crystallization ability and the large rate of crystallization affected the control of heat transfer during casting. The big morphology difference between the primary crystals of the CaO–SiO_2_–CaF_2_-based mold flux and the CaO–Al_2_O_3_-based mold flux is probably one of the biggest factors limiting lubrication between the CaO–Al_2_O_3_-based mold flux and high-Al steel during casting.

## 4. Conclusions

The effect of the cooling rate on the phase and crystal morphology transitions of a CaO–SiO_2_–CaF_2_-based mold flux and a CaO–Al_2_O_3_-based mold flux was investigated. The following conclusions were obtained:

1. Faceted cuspidine and CaSiO_3_ are co-precipitated when the cooling rate is less than 50 °C·min^−1^ in a traditional CaO–SiO_2_–CaF_2_-based mold flux. The different cooling rates can change the phases and crystal morphologies. The phases transform from Ca_4_Si_2_O_7_F_2_ and CaSiO_3_ to Ca_4_Si_2_O_7_F_2_ as the cooling rate increases from 30 °C·min^−1^ to 50 °C·min^−1^. Cuspidine shows four different morphologies: faceted shape, fine stripe, fine stripe dendrite, and flocculent dendrite.
2. CaAl_2_O_4_ and Ca_3_B_2_O_6_ are co-precipitated in the CaO–Al_2_O_3_-based mold flux. Neither the phases nor the crystal morphologies are altered as the cooling rate increases from 5 °C·min^−1^ to 50 °C·min^−1^. The morphology of CaAl_2_O_4_ was firstly dendritic, and then the dendritic morphology gradually changed to a large-mesh blocky shape at the cooling rates of 100 °C·min^−1^, 200 °C·min^−1^, and 500 °C·min^−1^. Different cooling rates do not show an obvious impact on the morphology transitions of CaAl_2_O_4_.
3. The strong crystallization ability and the large rate of crystallization of the CaO–Al_2_O_3_-based mold flux affect the control of heat transfer during casting. The big morphology difference between the primary crystals of the CaO–SiO_2_–CaF_2_-based mold flux and the CaO–Al_2_O_3_-based mold flux is probably one of the biggest factors limiting lubrication between the CaO–Al_2_O_3_-based mold flux and high-Al steel during casting.

## Figures and Tables

**Table 1:** The chemical compositions of the investigated mold fluxes.

Sample No.	Composition (mass%)					
	CaO	SiO_2_	Al_2_O_3_	Na_2_O	CaF_2_	B_2_O_3_
1	36	36	5	8	15	0
2	42	0	42	8	0	8
